# A point of view of poor control of diabetes: presence of disease and diagnosis time

**DOI:** 10.1186/1758-5996-7-S1-A167

**Published:** 2015-11-11

**Authors:** Fernanda Coelho, Gabriela Lopes, Luciane Souza, Erika Masanet Ripol

**Affiliations:** 1 Universidade Nova de Lisboa, Viçosa, Brazil

## Background

Developing countries has experienced changes in their profile, as the aging and alterations in lifestyle of population. It has been associated with the high increase in chronic diseases like Diabetes Mellitus Type 2 (DM2), which is connected with increasing prevalence of obesity and aging process. In Brazil, some studies have shown the difficulties of the health care to control diabetes as well as their complications. As a result, low quality of life (QL) is verified in the population with DM2, which may be the starting point to change the possible gaps on the treatment of the disease.

## Objective

The aim of this study was to evaluate the QL of adults and elderly with and without DM2. Two perspectives were considered to check QL: the presence and the time of diagnosis.

## Materials and methods

The survey population consists of individuals (men and women) with and without DM2, living in Viçosa-Minas Gerais, aged ≥ 40 yrs. and with low schooling. The sample was divided in three groups: control (CTL), individuals without DM2 or any disease in target organs; G1, individuals diagnosed DM2 ≥1 year and ≤5 yrs. and G2, individuals diagnosed DM2 ≥ 10 yrs. We checked sociodemographic and therapeutic data, cognitive state by Mini Mental State Exame, waist circumference (WC) and glycosylated hemoglobin (HbA1c). For the assessment QL SF-36v2® was applied.

## Results

198 individuals (CTL=81; G1=47; G2=61), with mean age 60.3±10.7 yrs. and schooling 4.7±2.9 yrs. were evaluated. 55.5% were elderly, 62.6% women and 57.3% using insulin. The cognitive state was similar between groups (CTL: 24.5±2.3; G1: 23.4±3.9; G2: 23±3.7). The WC showed significant difference between CTL and DM2 (p<0.001) with higher means to DM2 (104±15.7) compared to CTL (94.9±11.2). The HbA1C mean in both groups of DM2 was similar (G1: 9.1±1.9%; G2: 8.7±2.2%). Relating to QL, we found significant differences between CTL and DM2 just for physical component (p<0.001) without difference in mental component (p>0.05). Analyzing the time of diagnosis, no difference for QL were verified between G1 and G2 (p>0.05).

## Conclusion

People with DM2, in poor control of disease, presents significant lower QL when compared to CTL group. The physical component showed the strongest role in lower scores for QL. In the other hand, we not verify differences in QL as well as glycosylated hemoglobin between G1 and G2, suggesting that the diagnosis time could have been neutralized by poor control of DM2.

